# The Y498T499-SARS-CoV-2 spike (S) protein interacts poorly with rat ACE2 and does not affect the rat lung

**DOI:** 10.1099/acmi.0.000839.v3

**Published:** 2024-09-27

**Authors:** Amy L. Green, Dylan De Bellis, Evangeline Cowell, Roman V. Lenchine, Timothy Penn, Luke P. Kris, James McEvoy-May, Shailesh Bihari, Dani-Louise Dixon, Jillian M. Carr

**Affiliations:** 1 College of Medicine and Public Health, Flinders University, GPO Box 2100, Adelaide, South Australia 5001, Australia; 2 Flinders Health and Medical Research Institute, Flinders University, Adelaide, South Australia, Australia

**Keywords:** ACE2, inflammation, lentivirus pseudotype, rat, respiratory mechanics, SARS-CoV-2, spike protein

## Abstract

The rat is a useful laboratory model for respiratory diseases. SARS-CoV-2 proteins, such as the spike (S) protein, can induce inflammation. This study has investigated the ability of the Q498Y, P499T (QP-YT) amino acid change, described in the S-protein of the mouse-adapted laboratory SARS-CoV-2 MA strain, to interact with rat angiotensin converting enzyme-2 (ACE2) and stimulate responses in rat lungs. A real-time S–ACE2 quantitative fusion assay shows that ancestral and L452R S-proteins fuse with human but not rat ACE2 expressed on HEK293 (human embryonic kidney-293) cells. The QP-YT S-protein retains the ability to fuse with human ACE2 and increases the binding to rat ACE2. Although lower lung of the rat contains both ACE2 and TMPRSS2 (transmembrane serine protease 2) target cells, intratracheal delivery of ancestral or QP-YT S-protein pseudotyped lentivirus did not induce measurable respiratory changes, inflammatory infiltration or innate mRNA responses. Isolation of primary cells from rat alveoli demonstrated the presence of cells expressing ACE2 and TMPRSS2. Infection of these cells, however, with ancestral or QP-YT S-protein pseudotyped lentivirus was not observed, and the QP-YT S-protein pseudotyped lentivirus poorly infected HEK293 cells expressing rat ACE2. Analysis of the amino acid changes across the S–ACE2 interface highlights not only the Y498 interaction with H353 as a likely facilitator of binding to rat ACE2 but also other amino acids that could improve this interaction. Thus, rat lungs contain cells expressing receptors for SARS-CoV-2, and the QP-YT S-protein variant can bind to rat ACE2, but this does not result in infection or stimulate responses in the lung. Further, amino acid changes in S-protein may enhance this interaction to improve the utility of the rat model for defining the role of the S-protein in driving lung inflammation.

**Impact Statement**

While SARS-CoV-2 infection in humans can be managed in most cases, damaging and life-threatening inflammatory lung disease is still a problem. The rat is a widely used small animal laboratory model in which measuring lung function is feasible and, therefore, it is beneficial for studying the molecular and physiological basis for this inflammatory lung disease. A system in the absence of fully infectious SARS-CoV-2 enables experiments to be performed under routine biosafety containment conditions, and in a reductionist manner, to define the intrinsic ability of SARS-CoV-2 proteins as inflammatory stimuli. Our study has formed a basis for investigating S-protein in the rat lung which with further modification to improve ACE2 interaction and delivery of stimuli to the lung may clarify the mechanisms driving inflammation in the acute COVID-19 lung or persistent SARS-CoV-2 antigens that may contribute to long COVID.

## Data Summary

The authors confirm that all supporting data, code and protocols have been provided within the article or through supplementary data files.

## Introduction

Severe acute respiratory syndrome (SARS)-Coronavirus-2 (CoV-2) can infect the respiratory tract and cause coronavirus disease-2019 (COVID-19). This can be generalised into two phases: an early phase, involving approximately 3–7 days of viral replication with general cold-like or coryzal symptoms, and a later postviral inflammatory phase in the second week of illness. It is this second phase of the disease that presents the biggest risk for severe outcomes, such as respiratory distress and COVID-19 pneumonia [1,2]. Antiviral treatments are available to target the initial viral replication phase, whereas, the second phase of the disease requires respiratory support and management with anti-inflammatory strategies such as corticosteroid, or Tocilizumab, an anti-interleukin-6 receptor antibody. Anticoagulation therapy may also be clinically indicated, with these therapies recently reviewed [3]. Additionally, post-COVID-19 conditions, or long-COVID, are inflammatory pathologies that may reflect the persistence of SARS-CoV-2 RNA or proteins [4]. Our goal was to develop laboratory models to investigate the stimuli driving the COVID-19 inflammatory or thrombotic respiratory pathologies to better understand and manage the COVID-19 lungs.

The early ancestral SARS-CoV-2 variants did not infect rodents, such as laboratory mice [5]. One important restriction to infection was the poor interaction of the SARS-CoV-2 Spike (S) protein with the cell surface angiotensin II [angiotensin converting enzyme-2 (ACE2)] molecule in mice [6]. Seminal laboratory studies passaged ancestral SARS-CoV-2 in mice leading to the identification of S-protein variants in residues Q498P499-Y498T499 (QP-YT) that enhanced infectivity [5]. SARS-CoV-2 variants that later circulated in the human population such as alpha, beta and omicron, contain amino acid changes in the S-protein, including N501Y, that have been suggested to be the key to the interaction of S-protein with the mouse ACE2 molecule. Although ancestral SARS-CoV-2 is poorly infectious in rats, the alpha, beta and omicron variants can infect laboratory rats and cause lung pathology [7,8]. Rodent infection models with these SARS-CoV-2 variants are invaluable for laboratory research to define the viral phase of illness and aspects of replication associated with respiratory pathology. A simple, non-infectious system to define viral components contributing to inflammatory stimuli, thrombosis and respiratory dysfunctions would be of additional benefit, and the laboratory rat, as a long-standing animal, utilized for respiratory research [9] is a clear choice.

Studies have suggested that the S-protein alone or combined with LPS can induce inflammation in cells *in vitro* or in the lung [10,13]. Additionally, after the S-protein engages with ACE2 and a second protein, transmembrane serine protease 2 (TMPRSS2), the S-protein is cleaved to the S1 subunit. The S1 protein alone can induce inflammation in monocyte/macrophages, endothelial cells or when delivered into the lungs of mice expressing human ACE2 [14,16]. Following S-binding, ACE2 is internalised with downregulation at the cell surface. Low ACE2 levels are known to be associated with proinflammatory responses, loss of protective responses leading to lung injury and can impact the renin–angiotensin system [17,19]. Thus, here the interaction of the QP-YT variant S-protein with rat ACE2 was defined and further investigated for the ability to induce an inflammatory response using a strategy to engage ACE2 on a lentivirus pseudoparticle delivered intratracheally to the cells of the lower lung of the rat. Results demonstrate that the QP-YT mouse-adapted S-protein variant interacts with rat ACE2 *in vitro* but poorly and is not sufficient to infect or stimulate cells of the rat lung. Additional S-protein amino acid changes to improve binding to rat ACE2 may be required, or an alternative mode of delivery is needed to efficiently target the lung cells *in vivo*.

## Methods

### Cell lines

HEK293 (human embryonic kidney-293), 293T and HEK-ACE2 cell lines [20] were cultured in Dulbecco’s Modified Eagle Medium (DMEM) with 10% (v/v) foetal calf serum (FCS), penicillin (50 units ml^−1^), streptomycin (50 µg ml^−1^) and 1% (v/v) GlutaMAX at 37 °C in 5% CO_2_. Cell culture media and additives were from Gibco, Thermo Fisher Scientific. Human ACE2 m-Cherry was generated by transduction of HEK-ACE2 [20] with Vesicular stomatitis virus-glycoprotein (VSV-G) pseudotyped plenti6mCHERRY and selection with blasticidin at 5 µg ml^−1^ for 2 weeks, and then red fluorescent cells were selected by two rounds of fluorescence-activated cell sorting.

### Expression constructs

The constructs, referred to as L452R and QP-YT, were generated using the HDM-IDTSpike-fixK plasmid vector, expressing the S-protein from SARS-CoV-2 strain Wuhan-Hu-1 (termed ancestral S-protein) [20]. Site-directed mutagenesis was undertaken using the Q5 Site-Directed Mutagenesis Kit (New England Biosciences) and primers [L452R, Forward (F): CTACAATTATcggTACAGGTTGTTC and Reverse (R): TTTCTTGAAACCTTACTG] and (Q498P499-T498Y499, F: ATATGGTTTCtatacaACAAATGGAGTAGGGTATCAAC and R: GACTGGAGTGGAAAGTAG), with nucleotide changes indicated in lower case.

### Generation of HEK293 expressing mouse and rat ACE2, and its use in a fusion assay

Expression vectors for mouse (OMu22565D, NM_001130513.1) or rat (ORa01934D, NM_001012006.1) ACE2 were purchased (GenScript Biotech), and transfected into HEK293 cells, using Lipofectamine 3000 Transfection Reagent (Invitrogen). Stable transfectants were selected with Geneticin Selective Antibiotic (G418 Sulphate, Gibco) at a concentration of 500 µg ml^−1^. The cell lines were verified for ACE2 expression by immunostaining, and non-clonal stocks were stored. For fusion assay, 293T cells were co-transfected to express SARS-CoV-2 S-protein, as above, and a green fluorescent protein (GFP) reporter (pHAGE2-CMV-ZsGreen-W) using Lipofectamine 3000. During the 24-h post-transfection, cells were mixed with human, mouse or rat ACE2-bearing HEK293 cells, incubated for 24 h and imaged in real-time using the Incucyte SX5 Live-Cell Analysis System (Sartorius), using the 10× objective lens. Fused cells were identified by filtering for size > 500 µM and eccentricity in combination with green and/or red channel fluorescence. Quantitative outputs were measured as total fused cell area per image, averaged over five images per well, with *n* = 3–4 replicate wells per test sample.

### Generation of S-protein pseudotyped lentivirus

The S-protein pseudotyped lentivirus was generated using vectors and protocols adapted from Crawford *et al*. [20] with 1 µg of the lentiviral backbone [pHAGE2-CMV-ZsGreen-W (NR-52520)], 0.5 µg of the lentiviral packaging plasmid (psPAX2, Addgene #12260) and 0.25 µg of the viral envelope protein [either SARS-CoV-2 S (NR-52514), SARS-CoV-2 S with QP-YT mutation or VSV-G envelope (PMD2.G, Addgene #12259)]. An envelope-negative ‘no S-protein’ control was included. The lentivirus was harvested at approximately 72 h post-transfection, supernatant clarified, filtered (0.22 µM) and stored at −80 °C prior to use. For administration to rats, the lentivirus stocks were purified using 50 kDa molecular weight cut-off filters and centrifugation at 2500***g*** for 15 mins at 4 °C, prior to storage. Where indicated, the lentivirus was concentrated 10-fold by precipitation with a Lenti-X concentrator (Takara). The S-protein pseudotyped lentivirus was validated for infectivity on human HEK-ACE2 cells by quantitation of GFP-positive cells and particle number by PCR quantitation of the viral long terminal repeat (LTR) using primers (F: GTCTCTCTGGTTAGACCAGATCTG and R: TGCTAGAGATTTTCCAGACTGAC), as described below.

### S-lentivirus delivery to the rat lung and respiratory analysis

Sprague–Dawley rats (300–400 g; *n* = 21, total in the study) were anaesthetised, and control (media, *n* = 4 or no S-containing lentivirus, *n* = 2), ancestral (*n* = 2) or QP-YT (*n* = 3) S-protein pseudotyped lentivirus was delivered intratracheally in a volume of 200 µl, as previously described [21]. Lentiviral stocks used for the challenge of rats were approximately 0.5−1×10^4^ infectious units ml^−1^ and 1×10^6^ LTR copies ml^−1^. A low dose of LPS at 3 mg kg^−1^ (*n* = 3) was delivered intratracheally as a positive control. Rats were also administered lentivirus concentrated by Lenti-X precipitation, with *n* = 1 for each media, ancestral and QP-YT S-proteins or *n* = 2 ‘no-S-protein’ pseudotyped lentivirus. At 24 h post-administration, rats were anesthetised and intubated, and flexiVent respiratory mechanics were measured [21]. At the end of respiratory measurements, rats were humanely killed via exsanguination under isoflurane anaesthetic and a tracheostomy was performed with insertion of a 14-guage cannula into the trachea. The lungs and heart were removed *en bloc* and the lungs were lavaged with 32 ml kg^−1^ body weight 0.9% (w/v) NaCl, with each instillation withdrawn three times to generate a bronchoalveolar lavage (BAL). BAL cells were enumerated by light microscopy. Lung tissue was fixed in 10% (v/v) buffered formalin, and a section of the lower right-lobe was frozen in Trizol for RNA extraction.

### RNA extraction and reverse transcription quantitative polymerase chain reaction (RT-qPCR)

Tissue in TRIzol Reagent (Invitrogen, Thermo Fisher Scientific) was disrupted using a probe sonicator and total RNA was extracted with chloroform and isopropanol precipitated, according to the manufacturer’s instructions. Extracted RNA was DNase I-treated (New England Biolabs), and concentration and purity were measured (NanoDrop 2000 spectrophotometer). A total of 500 ng of RNA was reverse transcribed using 30 µM Random Primer Mix (New England Biolabs), 10 mM dNTPs (New England Biolabs), M-MuLV (100 units/reaction) (New England Biolabs) and RNase Inhibitor (8 units/reaction). RT-qPCR was performed on the resulting cDNA using PowerUp SYBR Green Master Mix (Applied Biosystems, Thermo Fisher Scientific) with 1 µM of each primer for rat cyclophilin/PPIa (F: CTTCGACATCACGGCTGATGG and R: CAGGACCTGTATGCTTCAGG), CXCL10 (F: ATGAACCCAAGTGCTGCTGT and R: CTCTCTGCTGTCCATCGGTC) and viperin (F: ACTATTTGGACATTCTTGCTATCTC and R: AATCAGGAGGCATTGGAAA). Real-time RT-qPCR was performed with a hot start protocol using a Rotor-Gene real-time PCR system (Qiagen) with the following settings: 1 cycle of 95 °C for 5 min, 40 cycles of 95 °C for 15 s, 59 °C for 30 s and 72 °C for 5 min, followed by a melt curve analysis. Results were normalised against the housekeeping gene, *cyclophilin*, and relative mRNA abundance determined by the Δ*Ct* method.

### Isolation of primary rat alveolar cells

Rats were humanely killed and lungs were removed, as mentioned above. Lung tissue was then digested with elastase at 0.5 mg ml^−1^ in balanced salt solution-B (BSS-B) [150 mM NaCl, 5 mM KCl, 10 mM HEPES, 0.1% (w/v) glucose, 2 mM CaCl_2_, 1.3 mM MgSO_4_ in 2.5 mM sodium phosphate and pH, 7.4] by syringe instillation of the fluid into the lungs and incubation at 37 °C for 45 mins fully submerged in 0.9% (w/v) NaCl. Lungs were then removed and minced with scissors/forceps in BSS-B containing DNase I, then incubated with FCS in a 37 °C water bath, with shaking for 2 mins. Lung tissue was serially filtered through 100, 40 and 30 µm cell strainers and the filtrate was centrifuged at 900***g*** for 10 mins at 4 °C. The cell pellet was resuspended in red blood cell lysis buffer (155 mM NH_4_Cl, 0.1 mM EDTA and 12 mM NaHCO_3_) and incubated at room temperature for 10 mins. Cells were pelleted, as above, and resuspended in DMEM with 10% (v/v) FCS and DNase I and were incubated for 20 mins at 37 °C on a petri dish. Non-adherent cells were removed, and adherent cells were fixed and immunostained for ACE2 and TMPRSS2 or cultured overnight in DMEM with 10% (v/v) FCS for lentiviral infection studies. *Ex vivo* infection studies were performed in two adherent lung cell isolations from *n* = 2 independent rats.

### Immunostaining for ACE2 and TMPRSS2

Fixed adherent alveolar cells were permeabilized [0.05% (v/v) IGEPAL (Sigma)], and non-specific binding was blocked [5% (v/v) human sera, 4% (v/v) normal goat sera and 0.4% (w/v) BSA in PBS]. Cells were immunostained with anti-ACE2 [rabbit polyclonal (20 µg ml^−1^) and Abcam (ab15348)] or anti-TMPRSS2 [rabbit polyclonal (20 µg ml^−1^) and Bioss (bs-6285R)]. Bound antibody was detected using goat anti-rabbit Alexa Fluor 555 (Invitrogen), and the nuclei were stained with Hoechst 33342 (5 µg ml^−1^, Thermo Fisher Scientific). Fixed tissue from the lower left lobe was paraffin embedded, sectioned and mounted on glass slides. Tissue permeabilization was achieved in EDTA buffer [1 mM EDTA, 0.05% (v/v) Tween-20 and pH, 8.0] at 95 °C for 30 mins. Non-specific binding was blocked by incubation in 10% (v/v) donkey sera in PBS, and the tissue was stained with anti-ACE2 or anti-TMPRSS2 antibodies, as above. Bound antibodies were detected using anti-Cy3 (Thermo Fisher Scientific), and nuclei were stained with Hoechst, as above. The tissue was mounted in buffered glycerol [0.5M Na_2_CO_3_ in 80% (v/v) glycerol and pH, 8.6]. Images were captured with epifluorescence microscopy using an Olympus IX83 inverted microscope (Olympus Life Science).

### Prediction of S-protein interaction with ACE2

Two-dimensional (2D) ACE2 protein sequences for *Homo sapiens* [(*H. sapiens*); accession: NP_001358344.1], *Mus musculus* [(*M. musculus*); accession: Q8R0I0.1] and *Rattus norvegicus* [(*R. norvegicus*); accession: NP_001012006.1], and TMPRSS2 protein sequences for *H. sapiens* (accession: NP_001369649.1), *M. musculus* (accession: NP_056590.2), *R. norvegicus* (accession: NP_569108.2) and *Mesocricetus auratus* (accession: XP_012971684.1) were obtained from the NCBI (National Center for Biotechnology Information) Protein database and aligned via Clustal Omega using Jalview [22]. The multiple sequence alignments were coloured according to Jalview percentage identity colour scheme, in which residues at each position are more strongly coloured if they share a higher percentage identity with the consensus sequence (https://www.jalview.org/help/html/colourSchemes/pid.html). Three-dimensional protein structures for the binding domains of ancestral S/human ACE2 (Protein data bank (PDB): 6M0J) [23], Omicron S/rat ACE2 (PDB: 8GRY) [6] and Omicron S/mouse ACE2 (7XOC) [24] protein complexes were retrieved from the Protein Data Bank (https://www.rcsb.org/). Models of rat ACE2 and the receptor binding domain (RBD) of SARS-CoV-2 QP-YT S-protein were generated via SWISS-MODEL [25] using the S-protein RBD from 6M0J as a template. Models of rat ACE2 binding to ancestral and QP-YT S-proteins were generated using HADDOCK v2.4 [26,27] and structures were imaged using PyMOL (Schrodinger, 2024). Amino acid residues critical for ancestral S/human ACE2 binding, as outlined in Lan *et al.* [23], were used to guide HADDOCK for the generation of both these structures and the subsequent HADDOCK scores. These residues are S-protein amino acids at positions 417, 446, 449, 487, 489, 493, 500, 501, 502 and 505, and ACE2 amino acid residues at positions 19, 24, 27, 28, 30, 31, 35, 37, 38, 41, 42, 45, 53, 79, 82, 83, 90, 353, 354, 355, 357 and 393. Polar contacts between these residues were determined. ACE2 residues that were not conserved between humans and rats were highlighted in teal. These amino acid residues are at positions 20, 24, 26, 27, 30, 34, 40, 59, 61, 64, 66, 67, 73, 74, 78, 79, 82, 83, 84, 91, 93, 95, 98, 335, 338, 339, 340, 342, 353, 359, 368 and 387.

## Results

### The QP-YT S-protein variant fuses with human, mouse and rat ACE2 expressing cells

**Fig. 1.**
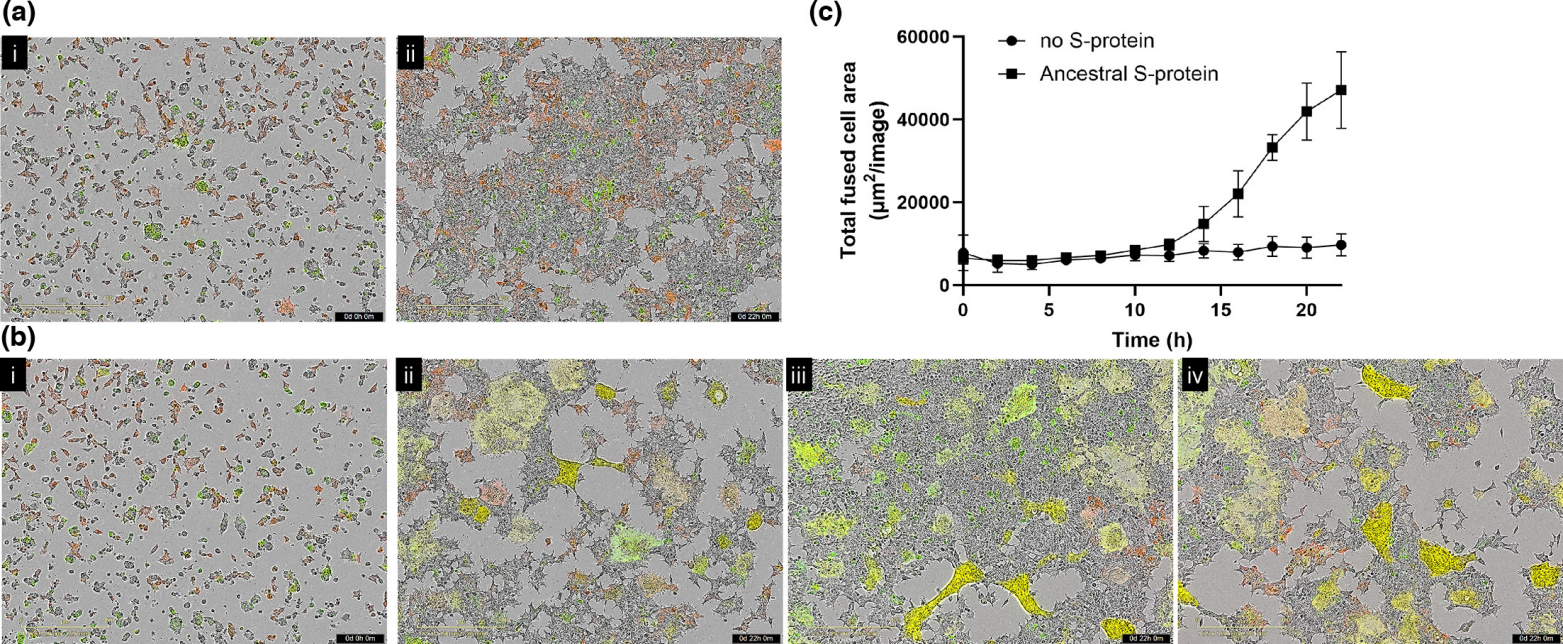
S-protein-ACE2 cell fusion. HEK293 expressing ACE2 and m-Cherry were plated and mixed with (a) 293T cells expressing GFP but no S-protein; or (b) 293T cells expressing GFP and ancestral S-protein. Cells were imaged (Incucyte, using 10× objective lens) with brightfield, green (wavelength) and red (wavelength) filters, every 15 min. Representative images are shown at (i) 0 h, immediately post-mixing and (ii–iv) 22 h post-mixing. Scale bar at bottom left-hand side = 400 µM; Time for post-mixing is indicated in the black bar at bottom right-hand side as days (d), hours (h) and minutes (m); and (c) fused cells were quantitated as red fluorescent protein (RFP)/GFP-positive, >500 µM from *n* = 4 replicates with *n* = 5 images per replicate; *p* < 0.001, Welch’s unpaired *t*-test with Holm–Sidak multiple comparisons test from 16- h post-mixing.

**Fig. 2.**
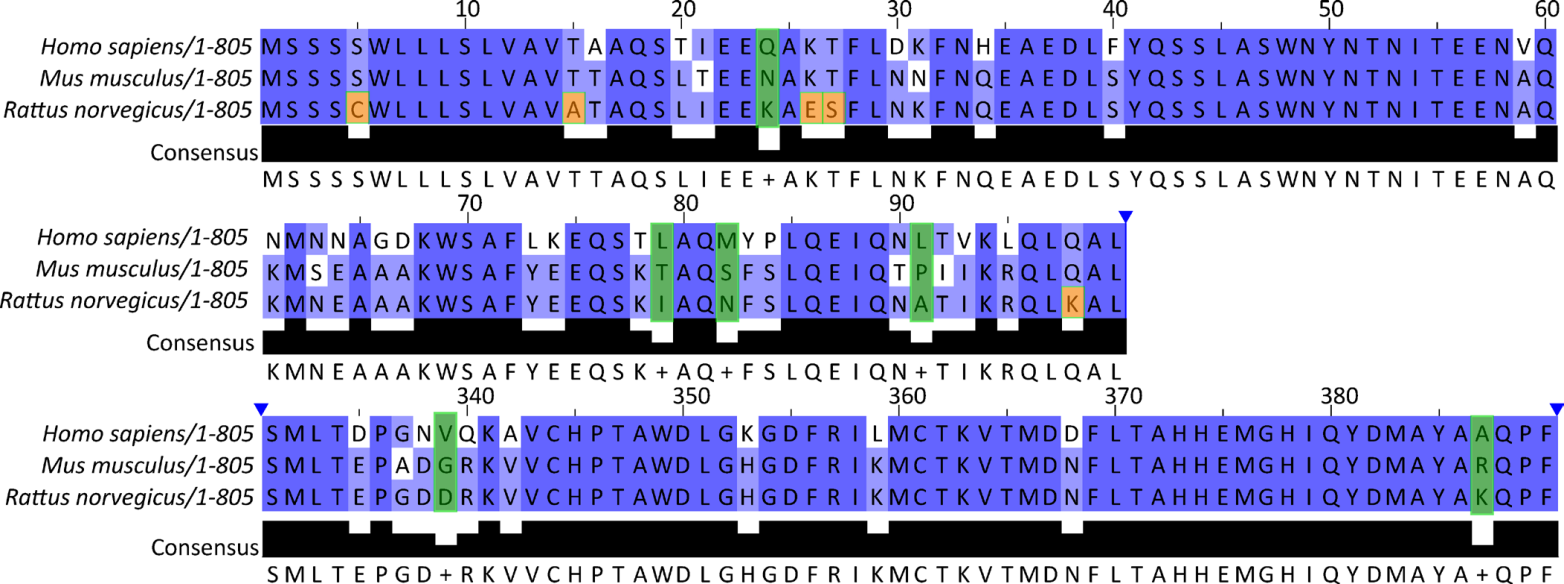
Amino acid sequence comparison of human, mouse and rat ACE2. ACE2 protein sequences for *H. sapiens*, *M. musculus* and *R. norvegicus* were obtained from the NCBI protein database and were aligned with Clustal Omega using Jalview. Amino acid residues 0–100 and 330–390 covering the S-protein interacting regions are shown. Green boxes indicate amino acid differences across all three species; orange boxes indicate amino acids that differ in rat ACE2 compared to mouse and human. Amino acid changes, T20L, D30N, H34Q and K353H are points of difference between human ACE2 that are conserved across mouse and rat ACE2.

**Fig. 3.**
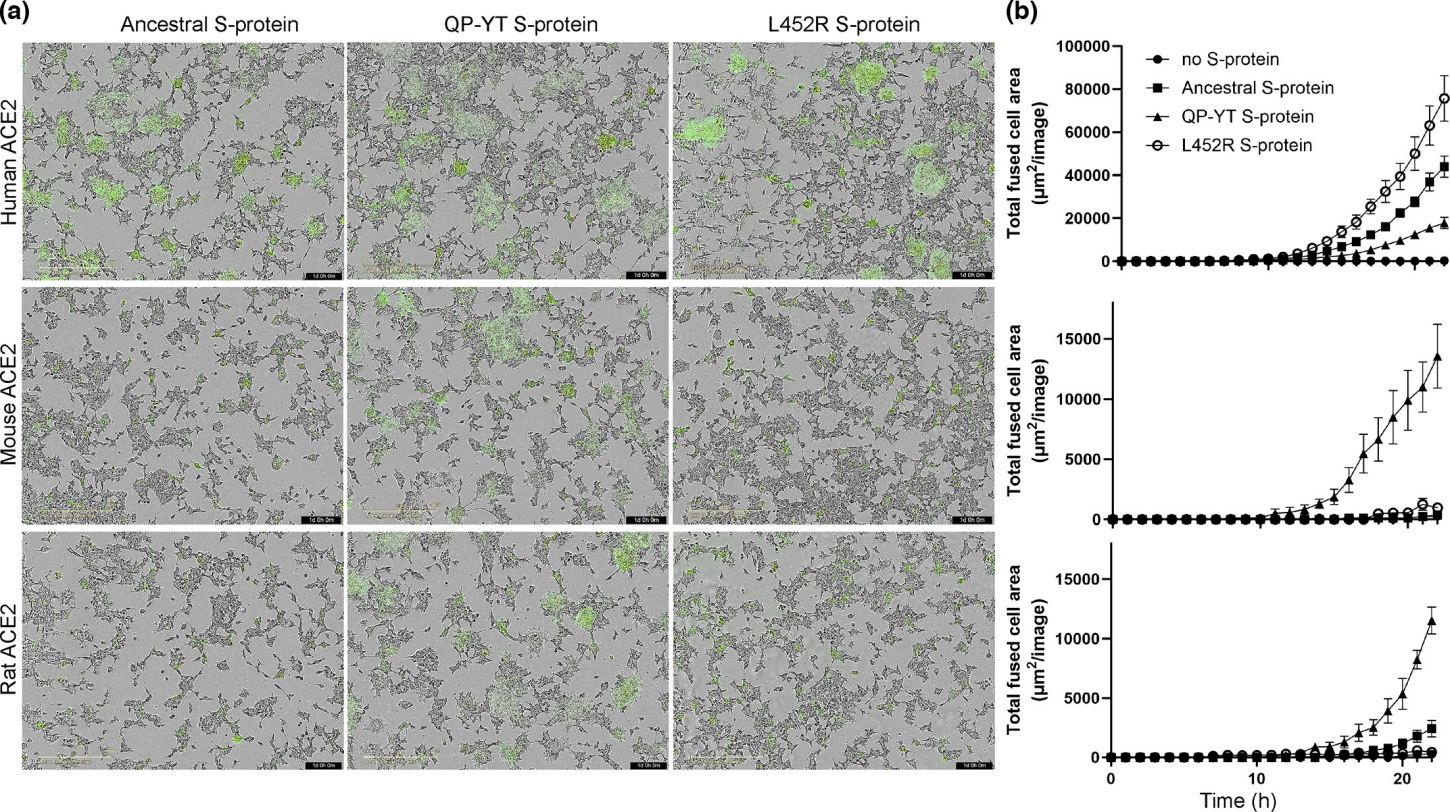
S-protein fusion with ACE2 from human, mouse and rat. HEK293 expressing ACE2 from human, mouse and rat were plated and then mixed with 293T cells expressing GFP (no S-protein) and ancestral, QP-YT and L452R S-proteins. Cells were imaged (Incucyte, 10× objective lens) with brightfield and green filters, every 15 min. (a) Representative images are shown 22 h post-mixing; the scale bar at bottom left-hand side = 400 µM; The time post-mixing is indicated as the black bar at the bottom right-hand side as days (d), hours (h) and minutes (m). Rendered videos are available at: https://www.youtube.com/playlist?list=PLBYayBF2_LCIDiYD0eJ8OJQIMuPD2vmSQ. (b) Total fused cells, defined as GFP-positive and >500 µM in size were quantitated and over time across the whole image, with five images averaged per well and *n* = 3 wells per treatment. Data were analysed by two-way ANOVA with multiple comparisons. Significant differences, with time and treatment, are described in the text.

To assess the binding and fusion of S-protein with ACE2, an assay was established whereby ancestral S-protein was co-transfected into 293T cells with a GFP-expressing construct. The transfected cells were mixed with a stable HEK293 cell line overexpressing human ACE2 and an m-Cherry reporter, and the fusion was imaged in real time. Representative fusion images demonstrate a visual S-protein and time-dependent cell fusion, with the mixing of the cell cytoplasm forming yellow fluorescence and large syncytia, which was confirmed by quantitative analysis (Fig. 1). Next, amino acid sequences of the interacting regions of ACE2 with S-protein were compared between humans, mice and rats(Fig. 2). Results demonstrate very few amino acid differences between mouse and rat ACE2, with key amino acid differences between human and mouse ACE2 at T20L, D30N, H34Q and K353H, also seen in rats. Based on this, it was predicted that the QP-YT mouse-adapted S-protein variant [5] may also bind to rat ACE2. Next, stable HEK293 cells overexpressing rat ACE2 and constructs to express ancestral, QP-YT mouse-adapted or L452R S-protein variants, the latter which reportedly stabilizes the S-protein [28] and is an amino acid change reported in the delta, which is infectious for rats [8], were generated. These were utilized in a similar fusion assay with 293T cells co-transfected with GFP. Green syncytia (>500 µM) were quantitated over time (Fig. 3). HEK293 stably expressing mouse ACE2 was used as a comparative control. Visually, the ancestral and QP-YT and L452R S-proteins variants were all fused with human ACE2 bearing HEK293 (Fig. 3a). In contrast, only the QP-YT variants showed any appreciable fusion with mouse or rat HEK293-ACE2 (Fig. 3a). Quantitation of GFP-positive syncytia demonstrated a significant increase in fusion compared to no S-control for all S-proteins from 12 to 14 h post-mixing and a larger fusion of L452R or ancestral than QP-YT S-protein with human ACE2 by 18 h post-mixing [*P* < 0.0001, *F*(Dfn, Dfd) = 27.88 (66, 264)], (Fig. 3b). The fusion of ancestral S-protein with human ACE2 was not significantly different to the fusion observed with L452R. For mouse and rat ACE2, quantitation demonstrated significantly increased fusion of QP-YT S-protein compared to no S-protein control from 17 to 18 h post-mixing (Fig. 3b). Ancestral S-protein demonstrated significant fusion with rat, but not mouse ACE2 at 22 h post-mixing compared to no S-protein, but was significantly less than that observed with QP-YT S-protein with rat ACE2 [*P* < 0.0001, *F*(Dfn, Dfd) = 22.53 (66, 276)]. No significant fusion was observed for L542R with either rat or mouse ACE2 (Fig. 3b).

### Rat lung tissue expresses both ACE2 and TMPRSS2 but does not respond to a S-protein pseudotyped lentiviral stimulus

**Fig. 4.**
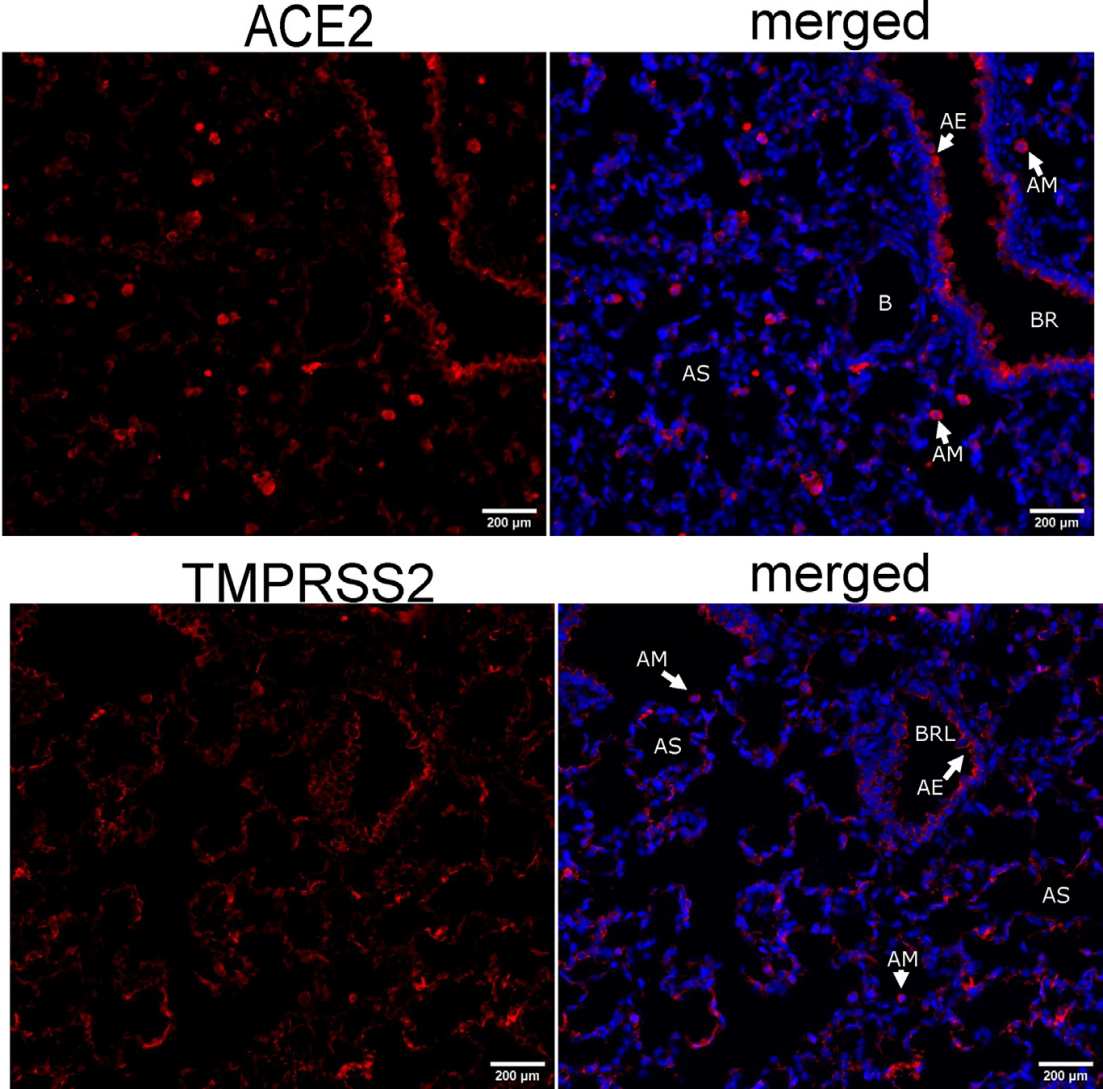
ACE2 and TMPRSS2 are expressed in cells of the rat alveoli. Lung tissue was collected from rats, fixed and immunostained for ACE2 and TMPRSS2. Nuclei were stained with Hoechst 333 442 and imaged by fluorescent microscopy. Images were captured at 20× magnification (Olympus IX83). Representative images are shown. The 200 µM scale bar is indicated and the structures annotated. **Abbreviations:** AE, airway epithelium; AM, alveolar macrophage; AS, alveolar space; B, blood vessel; BR, bronchi (large airway); BRL, bronchiole (small airway).

**Fig. 5.**
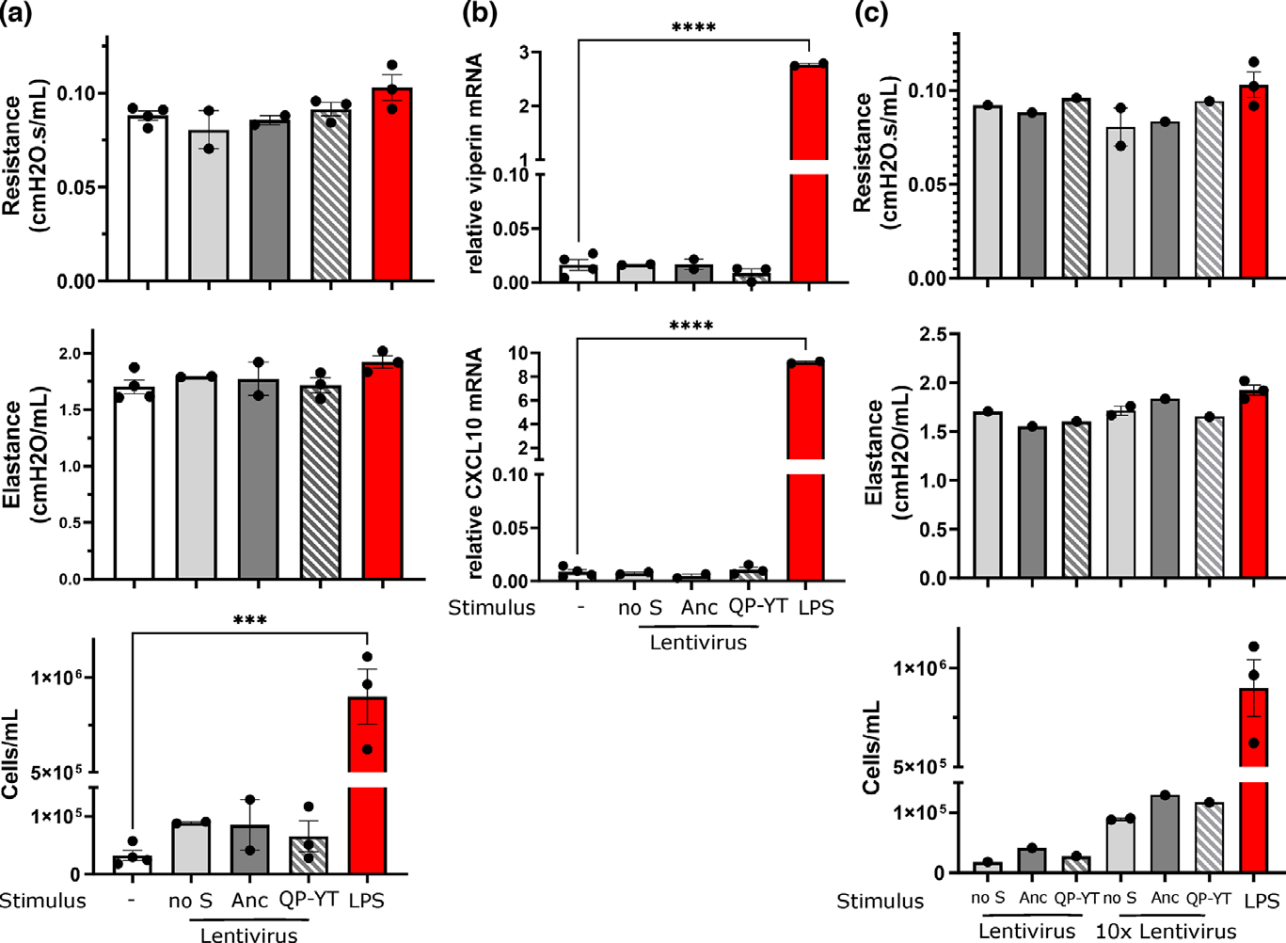
S-protein pseudotyped lentivirus does not affect the rat lung. Ancestral (Anc) or QP-YT S-protein pseudotyped lentivirus was intratracheally delivered to the rat lungs under anaesthetic. At 24 h following the delivery, rats were anaesthetized and (a) respiratory mechanics were measured (resistance and elastance). After termination of the experiment, the heart and lungs were excised under inflation, BAL was performed and cells were enumerated by microscopy (cells/ml); (b) RNA was extracted from lung lower-lobe tissue and subjected to RT-PCR for viperin and CXCL10, with normalization of results to cyclophilin. The points represent measures from individual animals: no stimulus (*n* = 4); no S-lentivirus (*n* = 2); ancestral S-lentivirus (*n* = 2); QP-YT S-lentivirus (*n* = 3); and LPS (*n* = 3). ***P < 0.005, two-way ANOVA with Tukey’s multiple comparison test; and (c) lentivirus was concentrated 10-fold by precipitation and delivered to rats as in (a) with *n* = 1–2 per treatment. The same LPS control is shown in (a) for comparison.

Since the QP-YT S-protein improved binding to rat ACE2, experiments next aimed to deliver this as a S-protein pseudotyped lentivirus to stimulate the rat lung. Expression of endogenous ACE2 and TMPRSS2 on cells of the respiratory epithelium and in the alveoli was observed confirming the presence of S-protein target cells in the rat lung (Fig. 4). Ancestral or QP-YT S-protein pseudotyped lentivirus was then administered intratracheally to anaesthetized rats. Controls included vehicle alone, a no S-containing lentivirus preparation or low-dose LPS (3 mg kg^−1^). Analysis at 24 h post-lentivirus stimuli saw no significant effect on respiratory mechanics, with resistance and elastance shown in Fig. 5a. Additionally, there was no induction of an inflammatory cellular infiltrate in the BAL (Fig. 5a). This contrasts with the cellular infiltrate measured in the BAL in response to the delivery of LPS (Fig. 5a). To assess the changes at a molecular level, lung RNA was extracted and subjected to RT-PCR for targets representing antiviral (viperin) and inflammatory products (CXCL10). No consistent induction or change in mRNA levels was associated with the delivery of ancestral or QP-YT S-protein pseudotyped lentivirus, while LPS control treatment induced both CXCL10 and viperin mRNA (Fig. 5b). Experiments were undertaken in a small cohort of rats treated with lentivirus concentrated 10-fold by precipitation. Similarly, there were no changes in respiratory mechanics (Fig. 5c). The cell numbers tend to increase in the BAL, but this was observed in all concentrated lentivirus preparations, including the no S-control, and hence is likely to be the precipitation reagent rather than a S-lentivirus-induced response (Fig. 5c).

**Fig. 6.**
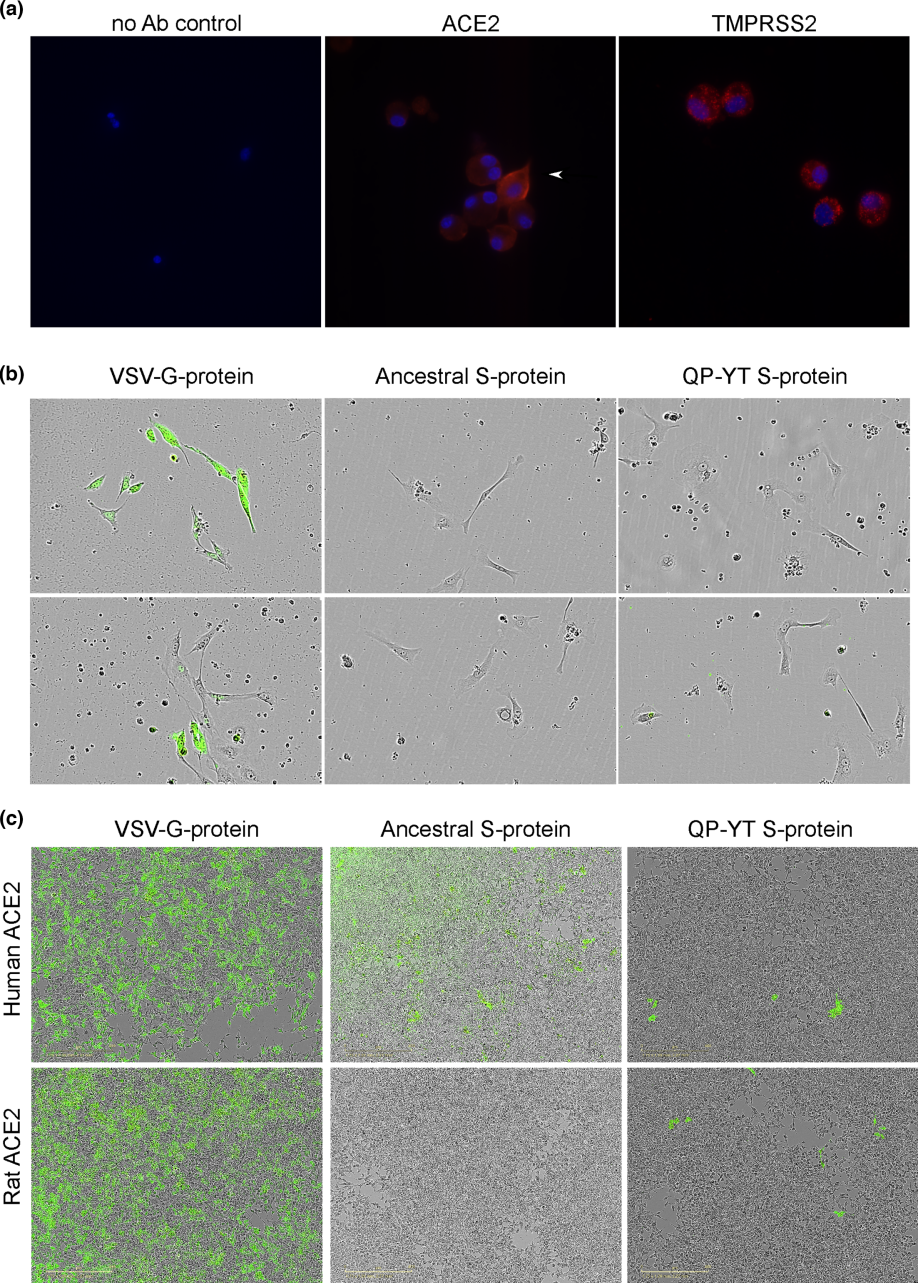
Adherent primary rat alveolar cells express ACE2 and TMPRSS2 but are not infected with S-protein pseudotyped lentivirus. Rats were anaesthetized, lungs extracted, digested and adherent cells (a) immunostained for ACE2 and TMPRSS2. Nuclei were stained with Hoechst 333 442. Images were captured by fluorescent microscopy at 40× magnification (Olympus IX83) and the representative images are shown. The white arrow indicates an example cell with intense ACE2 immunostaining; (b) cells were adhered, cultured overnight and challenged with VSV-G, ancestral or QP-YT S-protein pseudotyped lentivirus. The appearance of GFP-positive cells was monitored from 24 to 72 h post-challenge using the Incucyte, and two representative images from each treatment were exported; and (c) VSV-G, ancestral or QP-YT S-protein pseudotyped GFP reporter lentivirus was generated and used to infect human or rat ACE2 expressing HEK293 cells, and the appearance of GFP-positive cells visualized. Representative images are shown at 3 days post-lentiviral challenge (Incucyte, 10× magnification).

As no response was observed *in vivo*, experiments next isolated adherent alveolar cells from the rat lungs and assessed susceptibility to S-protein pseudotyped lentivirus challenge *ex vivo*. In freshly isolated adherent cells, immunostaining demonstrated the presence of ACE2 and TMPRSS2-positive cells (Fig. 6a). The cells were then adhered overnight and challenged with the pseudotyped GFP reporter lentivirus. Clusters of adherent cells were evident, and GFP-positive cells were readily detected with VSV-G control lentivirus as an amphotropic envelope pseudotype that will efficiently infect all cells, independent of ACE2 [29] (Fig. 6b). No GFP-positive adherent cell was detected in the ancestral or QP-YT S-protein pseudotyped lentivirus cells (Fig. 6b). Contaminating non-adherent cells were detected, and these could also appear GFP-positive. These were observed in both no S-control and GFP lentivirus-challenged cultures, including 0 h time-point controls (data not shown), and thus do not reflect S-lentivirus-infected cells. Pseudotyped GFP reporter lentivirus was next used to infect stable HEK293 cells expressing human or rat ACE2. S-protein pseudotyped lentivirus was poorly infectious compared to VSV-G pseudotyped lentivirus (Fig. 6c). Visual infectivity data align with the fusion assay outcomes with numerous GFP-positive cells with ancestral and fewer positive cells with QP-YT S-lentivirus infecting human ACE2-bearing cells. In contrast, GFP-positive cells, albeit few, were observed with QP-YT but not ancestral S-protein pseudotyped lentivirus in HEK293 cells expressing rat ACE2 (Fig. 6c). The same pattern of infectivity data was seen using Chinese Hamster ovary (CHO) cell lines overexpressing human or rat ACE2, demonstrating that this is not a cell line-dependent observation (data not shown).

Thus fusion of QP-YT S-protein occurs in rat ACE2-bearing cell lines. QP-YT S-protein pseudotyped lentiviral infection, however, occurs poorly in rat ACE2-bearing cell lines and not to any detectable levels in primary adherent cells isolated from the lower lung of the rat.

**Fig. 7.**
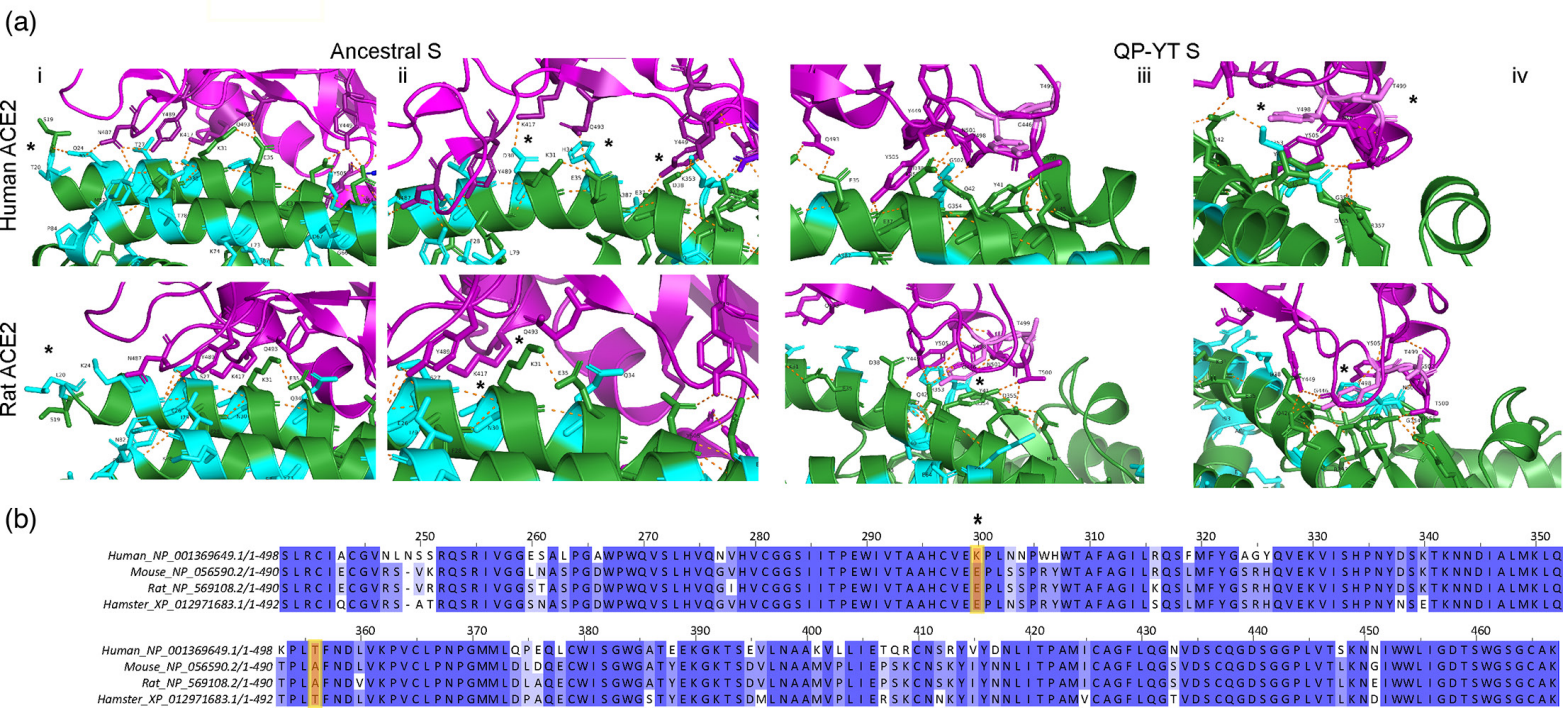
*In silico* analysis of the S-protein rat ACE2 interaction. (a) Predicted amino acid interacting interface of ancestral (i and ii) or QP-YT (iii and iv) S-protein with human or rat ACE2; (b) 2D alignment of human, mouse, rat and hamster TMPRSS2 from amino acid residues 240–470. * And orange box denotes sequence or structural regions of interest as discussed in the text. The teal colour indicates amino acid residues that vary between human and rat ACE2.

To predict ways to improve the interaction of S-protein with the rat, this was interrogated further through structural predictions of S-protein-ACE2. Ribbon structures of the RBD interface are shown for human and rat ACE2 interacting with ancestral or QP-YT S-protein (Fig. 7a). The T20L change in rat (and mouse) ACE2 positions the conserved S19 ACE2 residue differently to human, resulting in the predicted loss of several interactions observed between human ACE2 with amino acids at N487, Y489, K417 and Q493 on the ancestral S-protein [Fig. 7a(i)]. Similarly, the D30N and H34Q changes in rat and mouse ACE2 result in the loss of the interactions observed between the amino acids from human ACE2 with K417 and Q493, respectively, in ancestral S-protein. The QP-YT change was computationally introduced into the ancestral S-protein, and the interactions with human and rat ACE2 were predicted (Fig. 7a). Y498T499 is predicted to be positioned away from the human ACE2–S-protein interacting interface. In contrast, Y498 is predicted to be positioned in close proximity to Q42, as previously suggested in mice [5] and near to the aromatic ring structures of the amino acids in rat ACE2, in particular H353(Fig. 7a). The calculated Haddock scores indicate a lower-energy state for QP-YT (−150.1±1.2) compared to the ancestral S-protein (−137.6±1.4) or L452R (−137.2±2.1) interaction with rat ACE2, suggesting the QP-YT S-protein is a more favourable interaction for rat ACE2. Further, for binding and entry, the S-protein may be cleaved by the SARS-CoV-2 co-receptor, TMPRSS2. In our fusion assay and pseudotyped lentivirus infectivity assessment in cell lines (Figs3  6), the interaction may utilize human (HEK293) or hamster (CHO) TMPRSS2, but S-protein pseudotyped lentiviral infection of primary rat lung cells may utilize rat TMPRSS2. The 2D-sequence alignment of human, mouse and rat TMPRSS2 shows the conservation of amino acids at positions known to be important for interaction with S-protein, with the exception of K300E (Fig. 7b). This amino acid difference, however, is also observed with hamster TMPRSS2 (Fig. 7b), which is a SARS-CoV-2 susceptible laboratory animal, and fusion of S-protein pseudotyped lentivirus *in vitro* was also supported in CHO cells in our study (data not shown). Thus rat TMPRSS2 is unlikely to hamper with the S-protein pseudotyped lentiviral infection in rat cells.

## Discussion

The rat is a well-studied laboratory model for respiratory disease, but initial ancestral strains of SARS-CoV-2 did not infect laboratory rats or mice without modification to express human ACE2. Our goal was to utilize the rat as a research platform to define the triggers, characteristics and effectors of the COVID-19 lungs. The approach was to introduce a minimal amino acid changes on the backbone of ancestral SARS-CoV-2 S-protein, which is described to be inflammatory and pathogenic in humans and assess the effects of this on the rat lungs. Since the ancestral SARS-CoV-2, subsequent alpha, delta and omicron variants emerged and are reported to infect the Sprague–Dawley rats in the laboratory, with delta demonstrating the highest infection and lung pathology [8]. B1.351 (beta variant) can also infect the rat, with replication found mainly in the nasal turbinates and less in the lungs [30]. Alpha, beta, gamma and delta variants can infect cells bearing rat ACE2 *in vitro*, although the N501Y amino acid change showed no increased ability to interact with rat ACE2 in a fusion assay compared to ancestral S-protein [7]. Alpha (B1.1.7) can infect rats *in vivo*, although this is characterised by an acute infection with high levels of virus and lung pathology at day 2 post-infection and declining thereafter [7]. Alpha, beta, gamma and omicron contain the N501Y variation in the RBD that reportedly facilitates infection in mice. Delta does not contain N501Y; however, it does contain the L452R amino acid variation [31], suggesting N501Y is not essential for infection in the rat. Mouse-adapted SARS-CoV-2 variants initially described the Q498Y, P499T (QP-YT) amino acid changes, termed the MA strain [5], and subsequent adaptation to generate the MA10 strain with QP-YT and Q498K amino acid changes in the S-protein [32]. Yan *et al*. [33] also described 493R and 501T variants arising on passage of the MA strain in mice that suggested interaction with N31/Q34 and H353, respectively, of mouse ACE2. A lentivirus system to express mouse ACE2 with amino acid changes at N31K and H353K to reflect human ACE2, increased SARS-CoV-2 infection. This, however, may be improved up to 58% of the replication compared to human ACE2, by further changes in N30D and F83Y, suggesting multiple changes are required to facilitate efficient S-binding to mouse ACE2 [34]. Similarly, studies have humanized the mouse ACE2 receptor by clustered regularly interspaced short palindromic repeats (CRISPR)-editing of the H353K change or in combination with S82M and F83Y to facilitate infection [35,36]. Interestingly, a 498 h variant in combination with E484D was observed with the latter conferring ACE2-independent infection in mice [33].

Considering both the rat and mouse tropism data above, our study chose to assess amino acids selected by passage in mice (QP-YT), and L452R as shown in the delta.

A laboratory fusion assay was established, as has previously been published [28,37,40]. The fusion assay reflects parts of the entry process: functional S–ACE2 binding, cleavage of S by TMPRSS2 and S2 fusion to the cell surface [41]. Our assay utilized a fluorescent visual assessment of cells and syncytia formation, rather than luminescence outputs [28,42]. Results indicated that the L452R variant did not improve the interaction with rat ACE2. QP-YT S-protein was less effective in fusing with human ACE2 than ancestral or L452R, but QP-YT did facilitate fusion with both mouse and rat ACE2. Since the interaction and fusion of S-protein with rat ACE2 can be afforded by the QP-YT change alone, QP-YT S-protein pseudotyped lentivirus was generated which infected human and rat ACE2 expressing cell lines, in a manner consistent with the fusion assay, although poorly. Our structural analysis predicted an interaction of Y498 in S-protein with H353 in rat ACE2 to form a presumptive favourable pi–pi ring-stacking interaction, with a predicted lower-energy state. The co-receptor TMPRSS2 also plays a role in S-protein cell fusion. Our data demonstrate S-fusion in rat ACE2-bearing cells of human and hamster origin, and amino acid sequence comparison demonstrated no changes across the proposed S–TMPRSS2 interacting regions in rat compared to human and hamster, suggesting species-specific TMPRSS2 is not a likely barrier to fusion here. However, no QP-YT S-protein pseudotyped lentiviral infection was seen in primary rat cells isolated from the lower lung of the rat. Interestingly, this is similar to a previous report where B1.1.7 (alpha)-infected rat nasal turbinates, but did not replicate well in the lower rat lung [30]. In our study the expression of both ACE2 and TMPRSS2 was detected in tissue from the lower rat lung as well as primary cells extracted from the alveoli, likely representing alveolar macrophages, supporting the presence of ACE2-bearing target cells in the rat lung.

Our goal was to study individual drivers of respiratory or inflammatory dysfunction of SARS-CoV-2, and similar studies have been undertaken with the SARS-CoV-2 M protein delivered as protein stimuli to the mouse respiratory tract [43] or open reading frame (ORF)7b protein, delivered by an Adeno-associated vector (AAV)-9ORF7b expressing vector [44] or using S-protein delivered to mice expressing human ACE2 [45]. Pathogenic or inflammatory responses to the S-protein in the lung are also supported by studies showing that S1, a cleavage product of S following ACE2 engagement, can activate nerve cells in an *ex vivo* tracheal-lung model [46] and activate monocyte inflammatory responses *in vitro* [47]. S-protein can also directly impact endothelial cell barrier function via various processes, which can be independent of ACE2 and involve TGF or integrin signalling [13,48]. In the study of Gu *et al*., S-protein was co-administered with poly I:C and induced a rapid inflammatory response within 24 h in the context of human ACE2 expressed in the lungs of mice [45]. Our study had aimed to similarly use a dsRNA response from the incoming lentiviral vector, alongside S-protein stimulus with the benefit being the natural distribution of ACE2/TMPRSS2 on the rat lung target cells and engagement of ACE2 by the lentiviral particle. In this context, however, our study did not observe induction of an inflammatory response. The lack of inflammatory effect following QP-YT S-protein pseudotyped lentivirus delivery to the lung in our study may be due to the poor targeting of rat ACE2. Our *in silico* predictions of the interaction of rat ACE2 with ancestral and QP-YT S-proteins suggest the Y498 residue, in particular, improves the interaction with S-protein, but changes at other positions in the ancestral S-protein, such as N487 or even F486V as observed in alpha and omicron, can alter the impact of the T20L change in rat ACE2 and improve protein interactions at this region of the S–ACE2 interface, as suggested from studies in mice [33,49, 50]. Similarly, K417N, as observed in alpha and omicron, can improve the interaction with N30 in rat and Q493R or K arising in mouse-adapted S-protein variants [32,33] can improve interactions with rat Q34. Prior *in silico* predictions of S-interactions with rat ACE2 have proposed interactions of S-protein at positions 477/478 with rat ACE2 at K24; 452 and 486 with Q34 and N82, respectively, and 501/498 with H353 of rat ACE2 [8]. Our study supports the importance of the Y498–H353 interaction, but suggests that the L452–Q34 interaction, at least in isolation, is not sufficient to confer the ability of the S-protein to interact with rat ACE2. The importance of the R493Q variant in omicron in facilitating cross-species ACE2 interactions has been suggested [6]. While S-protein from omicron variants can be incorporated into this system, it is unclear if this form of S-protein still retains pro-inflammatory properties, with the overall infection in laboratory animal models showing less pathology in the lung [51]. Although no longer circulating in the human population, the S-protein from the delta variant, which effectively infects and causes pathology in rats is also a promising future amendment to study.

Additionally, prior literature and our observations suggest that the S-protein pseudotyped lentivirus is poorly infectious compared to the VSV-G pseudotyped lentivirus. Hence, S-lentivirus may not be stable in the lung environment. L452R is not present in the S–ACE2 interacting interface and this alone did not increase S-protein interaction with rat ACE2, but reportedly stabilizes the S-protein [28] and may be of benefit when combined with QP-YT S-protein to improve the stability of the S-protein pseudotyped lentivirus. Further, lentivirus access to the lung surface is known to be difficult and may be impeded by lung secretions. Methods have been developed to increase the efficiency of lentivirus transduction of the lung, for instance, by treatment with agents such as lysophosphatidylcholine [34,52]. This type of methodology does damage the lung, for instance with epithelial cell disruption [52], and can impact the cells we are aiming to target as the important components of the inflammatory pathology of COVID-19. Other alternative delivery vectors such as AAV, as utilized for ORF7b in mice [44], may be useful in delivering other SARS-CoV-2 proteins to the lung in the future. To study S-protein, however, the AAV genome cannot accommodate full-length S-protein, but other viral pseudoparticle delivery systems such as VSV, and incorporating a *C*-terminal deletion of S-protein may be appropriate [53].

In conclusion, the S-protein QP-YT variant can engage rat ACE2 *in vitro* but does so relatively poorly. The rat ACE2 and TMPRSS2 are present in the lower lung, however, QP-YT S-protein pseudotyped lentivirus does not deliver a stimulus that induces any measurable inflammatory or respiratory changes in the rat and further amino acid changes to adapt S-protein to rat ACE2, or alternative delivery systems to the rat lung are needed.

## Abbreviations

AAV: adeno associated virus
ACE2: angiotensin converting enzyme-2
BAL: bronchoalveolar lavage
CHO: chinese hamster ovary
COVID-19: coronavirus disease-2019
2D: two dimensional
DMEM: dulbecco modified eagle medium
F: forward
FCS: foetal calf serum
GFP: green fluorescent protein
HEK293: human embryonic kidney-293
H. sapiens: Homo sapiens
LPS: lipopolysaccharide
LTR: long terminal repeat
M. musculus: mus musculus
ORF: open reading frame
QP-YT: Q498P499-Y498T499
QP-YT: Q498Y, P499T
R: reverse
RBD: receptor binding domain
RFP: red fluorescent protein
R. norvegicus: Rattus norvegicus
RT-qPCR: reverse transcription quantitative polymerase chain reaction
S: spike
SARS-CoV-2: severe acute respiratory syndrome coronavirus-2
TMPRSS2: transmembrane serine protease 2
VSV-G: vesicular stomatitis virus glycoprotein
